# Development and validation of the prenatal activity restriction stress questionnaire: a Rasch rating scale analysis

**DOI:** 10.1186/s12884-020-03347-3

**Published:** 2020-10-31

**Authors:** Hsiao-Ying Hung, Su-Pin Hung, Ying-Ju Chang

**Affiliations:** 1 Department of Nursing, College of Medicine, National Cheng Kung University, Tainan, Taiwan; 2 Center of Teacher Education & Institute of Education, National Cheng Kung University, Tainan, Taiwan; 3 Institute of Allied Health Sciences & Department of Nursing, College of Medicine, National Cheng Kung University, Tainan, Taiwan; 4 Department of Nursing, National Cheng Kung University Hospital, Tainan, Taiwan

**Keywords:** Antepartum activity restriction, Preterm labor, Prenatal stress, Rasch analysis, Instrument development

## Abstract

**Background:**

Antepartum activity restriction (AAR) is a common recommendation given to women at risk for preterm delivery. However, such treatment has been shown to cause heavy emotional burdens on the women receiving it since it requires them to face many challenges derived from the intervention. Nevertheless, current existing scales lack effective items that can reflect the distress of these women. The aim of this study was to develop a reliable instrument to assess the distress of women experiencing AAR.

**Method:**

The Prenatal Activity Restriction Stress Questionnaire (PARSQ) was developed according to comprehensive literature review, women’s interviews, and existing pregnancy-special stress scales from August 2016 to July 2017 in southern Taiwan. Six experts evaluated its content validity; the Rasch rating scale model (RSM) was used to examine its item-fit, dimensionality, and reliability with 200 women with AAR experience. Furthermore, the concurrent validity was assessed through computing the correlation of AAR women’s scores on the PARSQ and Perceived Stress Scale (PSS), and discriminant validity of the PARSQ was assessed to compare the scores’ differences between the AAR women and the healthy pregnant women.

**Results:**

The PARSQ was constructed with 23 items in the 4-dimensional scale: Role function changes (8 items), Fetal safety and health (5 items), Physical and psychological care issues (5 items), and Socioeconomic and medical issues (5 items). It was confirmed to have satisfactory content vitality (CVI = 0.78 to 1.0), reasonable item-fit (0.77 to 1.51), and good reliability in RSM model, as well as adequate concurrent validity (*p* = 0.005) and discriminant validity (*p* < 0.001).

**Conclusions:**

Understanding the distress of women undergoing AAR is necessary for developing appropriate prenatal care to *assist women* in coping with their situation to alleviate their emotional burdens. The developed PARSQ with satisfied psychometric properties can be an informative instrument for clinicians/researchers to assess the specific stress of pregnant women with AAR.

**Supplementary Information:**

The online version contains supplementary material available at 10.1186/s12884-020-03347-3.

## Background

Preterm birth has been known to seriously threaten the health of newborns. Therefore, finding solutions to strengthen the prenatal care of women to avoid preterm birth is a global endeavor [1].

Antepartum activity restriction (AAR) is a common recommendation prescribed for women with threatened preterm labor (TPTL) [2], which is the main precursor and the most common cause of treatment or hospitalization during pregnancy [3]. Whether they are in the hospital or at home, women are advised to implement AAR to avoid the worsening of their symptoms [4] since AAR is thought to calm uterine activity and increase blood flow of the uterus, which may benefit the health of the mother and the fetus [5].

Although AAR is intended to alleviate women’s TPTL symptoms to decrease their risk of preterm birth, previous qualitative studies have shown that women were required to cope with many challenges derived from it. To respond to activity restriction, women must suspend their work, redistribute their roles in the family, learn to monitor the subtle changes in their bodies, and adapt to the side effects of tocolytics. Such living contexts change a woman’s self-identity and cause heavy emotional burdens [6, 7]. Mounting evidence has shown that woman’s negative prenatal emotions can lead to adverse pregnancy outcomes, like preterm birth and low infant birth weight [8, 9], and negative consequences of maternal and offspring health [10]. Therefore, the prenatal emotional status of women undergoing AAR requires greater attention.

To date, several types of questionnaires have been applied to measure women’s prenatal emotional status. The first type measures stressful life events, such as unemployment or divorce, that occur during pregnancy (e.g., the Life Events Inventory [11]); the second assesses the general emotional status of pregnant women (e.g., the Perceived Stress Scale [12] and Edinburgh Depression Scale [13]); and the third type evaluates the pregnancy-specific stress that arises from the women’s concerns about physical condition and changes, baby health, and upcoming labor (e.g., the Prenatal Distress Questionnaire [14] and Pregnancy-Related Anxiety Scale [15]). A recent meta-analysis has shown that pregnancy-specific stress measures had greater predictability for women’s adverse pregnancy outcomes than other kinds of psychological stress measures [8].

Although 15 pregnancy-specific stress questionnaires have been developed [16], most were based on the context of healthy pregnant women [17, 18]. Accordingly, those scales seem more suitable for healthy pregnant women than high-risk pregnant women. Although one scale has been developed based on the life experiences of medically high-risk pregnant women [19], it lacks certain items reflecting the distress of women undergoing AAR due to their TPTL symptoms, such as issues of drug treatment and finding substitutes for their family and workplace functions [6, 20]. Consequently, the stress of the women undergoing AAR cannot be reflected fully, which impedes the development of appropriate care measures for assisting these women in coping with their situation to alleviate their stress. In response, this study aimed to develop an instrument that effectively measures the distress or concerns of women undergoing AAR.

## Methods

Following an instrument-development procedure [21], three-stage development of the instrument was conducted in southern Taiwan from August 2016 to July 2017 with approval by the Institutional Review Board (A-ER-105-001). In the first stage, a comprehensive literature review and interviews of Taiwanese women with AAR were conducted to conceptualize “Prenatal Activity Restriction Stress,” and then items were generated and modified according to this definition to develop the initial Prenatal Activity Restriction Stress Questionnaire (PARSQ). The second stage involved expert panels examining the content validity of the initial PARSQ. In the third stage, the psychometric properties of the modified PARSQ were tested.

### Initial PARSQ development

Firstly, a series of literature findings exploring the experience of women undergoing AAR due to their TPTL were reviewed. These studies revealed that women were burdened by both their TPTL and activity restrictions. At the moment of being diagnosed with TPTL, a woman’s joyful and healthy pregnancy may suddenly change into an abnormal pregnancy filled with fears and worries. It also raises the woman’s concerns about fetal health and their own physical and psychological discomfort. Activity restrictions prescribed for preventing preterm birth force these women to alter their original lives drastically, like being suspended from work and not being able to complete their original roles in the family. Moreover, they have had to monitor all the subtle changes in their bodies. The huge lifestyle changes can cause heavy emotional burdens for these women [6, 7, 20, 22–24].

The literature review findings were then used as the fundamental knowledge to develop the interview guide to interview individually 10 Taiwanese women with AAR due to their TPTL. Most of them were under age 40 (*n* = 9), and half had experienced hospitalization for treating their TPTL symptoms (*n* = 5). These women reported that their AAR at home lasted for at least 14 days, and all had experience of taking tocolytic drugs for controlling their TPTL. The interviewed women reported that *they too had faced the* challenges *and conflicts between their inactivity, family life and* career similar to the findings of the literature review.

Findings from the literature review and the Taiwanese women’s interviews were then integrated to define the construct of “Prenatal Activity Restriction Stress” as 4 dimensions of pregnant women’s distress or concerns due to their activity restrictions and pregnancy complications: (1) Role and function changes; (2) Physical and psychological care issues; (3) Fetal safety and health; and (4) Socioeconomic and medical issues. Afterwards, the items were generated according to this definition and other existing stress scales addressing women with high-risk pregnancies [14, 15, 19, 25]. The initial version of the PARSQ containing 29 items with a 5-point scale ranging from 1 (never) to 5 (always) was developed.

### Content validity of the initial PARSQ

The content validity of the initial PARSQ was evaluated by six experts with specialties in maternal health, research, and questionnaire development. An item-review document containing a cover letter with an explanation of the PARSQ’s purpose and an item-scoring form with instructions was mailed to the panel of experts who were asked to evaluate the relevance, correctness, and comprehensibility of the PARSQ items with a 4-point scale ranging from 1 (not appropriate) to 4 (very appropriate), and to provide suggestions for revising the items. The relevance of items means the degree to which the items relate to a particular construct; the correctness of items means the degree to which they guide respondents toward providing genuine answers, and the comprehensibility refers to the degree to which the item’s wording is easy to understand. Subsequently, the content validity index for each item (I-CVI) and the content validity index for all scales (S-CVI) were computed. The I-CVI is calculated as the percentage of experts giving either 3 or 4 on a 4-point scale, while the S-CVI is the average of the total I-CVI in the scale. I-CVI values equal to or greater than 0.78 and an S-CVI greater than 0.8 are considered appropriate when there are six or more experts rating the items [26]. The ranges of the CVI of relevance, correctness, and comprehensibility for each item on PARSQ were 0.67–1.0, 0.67–1.0, 0.5–1.0, respectively, with four items (items 4, 5, and 11–14) demonstrating unacceptable CVI (Additional file 1) due to unclear and redundant content commanded by the experts. After the four items were modified, the remaining 25 items were then processed for further psychometric properties testing.

### Psychometric properties examination of the modified PARSQ

A total of 200 women with AAR experience and 96 healthy pregnant women participated in examining the psychometric properties of the modified PARSQ.

### Participants

The target group of this study was women with AAR experience either in the hospital or at home. The eligibilities of the AAR women were being at least 20 years of age, having AAR prescribed or recommended by their doctors due to their TPTL symptoms, such as preterm uterine contractions or vaginal bleeding, before 37 weeks of pregnancy within 1 year. Their responses on the modified PARSQ were analyzed for item quality, dimensionality, concurrent validity, and reliability of the instrument. The healthy pregnant women were at least 20 years old and with no pregnancy complications and AAR experience. Their responses on the modified PARSQ were used to examine the instrument’s discriminate validity. Women in both groups who had ever been diagnosed with depression or other affective disorders were excluded.

Purposive sampling was used to recruit the participants in a medical center in southern Taiwan. The participants were recruited mainly via doctor and case manager referrals when attending their regular antenatal or postpartum check-ups at an outpatient clinic. Since few studies lay down substantive rules for determining the sample size calculations for the Rasch model and how to calculate the power of a test power, a rule-of-thumb guide of at least 10 observations per category of scales for reaching the precision of the estimates was used [27]. The responses in the PARSQ category in this study meet this criterion.

### Instruments

#### Prenatal activity restriction stress questionnaire (PARSQ)

The PARSQ is a self-reported scale designed to detect four domains of specific distresses of activity restriction, including (1) Role function changes; (2) Fetal safety and health; (3) Physical and psychological care issues; and (4) Socioeconomic and medical issues. The initial PARSQ contained 29 questions, which were reduced to 25 after the expert review. Moreover, to account for variations in the situation of each woman, six filter questions (items 3, 5, 15, 18, 23, 24) that attempted to explore the issues of childcare and employment were included. If a respondent had not experienced what the filter question described, they could skip the question. Items in the PARSQ were rated on a 5-point scale from 1 (never) to 5 (always), with higher scores indicating higher distress.

#### Perceived stress scale (PSS)

The Chinese version of the 10-item PSS with adequate reliability and validity [28] was adopted in this study. It is a self-reported questionnaire originally developed by Cohen and Williamson (1988) [29] for assessing the general perceived stress status of people in a given situation within the past month. The items on the PSS-10 were rated on a 5-point scale ranging from 0 (never) to 4 (very often), with higher scores indicating higher perceived stress.

### Data analyses

Although test models of Classic Test Theory (CTT) have been applied for decades in developing measuring instruments due to its simple assumptions, the main shortcomings of CTT are both a person’s true scores and item parameters (e.g., item difficulty and item discrimination) that are test- and sample-dependent [30, 31]. To address the disadvantages of CTT, many IRT test models, including the rating scale model (RSM), were developed [32]. The RSM, a Rasch-family model, identifies responses in scales with a fixed set of rating points by using complex mathematical equations to estimate the probability of a person endorsing a specific response category of a given item. This method can not only overcome the deficiencies of CTT but also can determine the problematic items through analyzing people’s aberrant responses to them [33, 34]. Due to the advantages of the RSM, the RSM combined with CTT was applied in examining the psychometric properties of the PARSQ. Several methods, including item analysis, dimensionality, reliability, interpretability, differential item functioning (DIF), and concurrent and discriminant validity, were adopted.

### Item analysis

Descriptive statistical analysis, extreme groups’ analysis, and RSM, was performed to evaluate the quality of each item on the modified PARSQ. In descriptive statistical analysis, the mean scores and standard deviation of each item are analyzed. If an item has an obviously high or low mean score or low standard deviations (≤ 1.0), it indicates that modification or deletion of the item should be considered because it may be too hard or too easy for the respondents or has low discrimination [35].

Extreme groups’ analysis was used to determine whether an item can differentiate respondents with high scores from those with low scores [36]. During the computation of this analysis, the upper and lower 27% of the scorers of each item were identified and then sorted into high- and low-score groups. Thereafter, independent *t*-tests were used to test for significant differences between these two groups, where significance indicates acceptable discrimination.

RSM with two mean-square fit statistics (MnSq) weighted (Infit) and unweighted (Outfit) were adopted to determine undesired response patterns and problematic items [34]. The Infit is an indicator used to detect aberrant response patterns on items near the ability level of the respondents, while Outfit is sensitive to unexpected response patterns on items far exceeds respondents’ ability. The ideal values of Infit and outfit are 1.0, which indicates little distortion in the instrument. Generally, the acceptable range of an item’s Infit and Outfit is 0.5–1.5 [37]. After poor-quality items were deleted based on the item-analysis results, the PARSQ’s validity and reliability were then evaluated.

### Dimensionality, reliability, interpretability, and DIF evidence

Unidimensional RSM and multidimensional RSM were applied to verify the structure of the PARSQ. The deviance difference between these two models was approximated by a Chi-square distribution with degrees of freedom, which is the difference in the estimated number of the two models, to determine which hypothesized model was the best-fit [38]. After confirming the PARSQ’s structure, the item-separation reliability and person reliability (expected a posteriori/plausible value, EAP/PV) were obtained to support the PARSQ’s reliability. Item-separation reliability reflects whether the questionnaire items can effectively separate respondents with different levels of latent ability; whereas person reliability is the reproducibility of respondents’ abilities when they respond to a parallel test, which is equivalent to the concept of consistent reliability (Cronbach’s alpha) in CTT. The acceptable value of item and person reliability is 0.6–0.8 [39]. Finally, a visual graph representing the distribution of item and person locations was illustrated to support the PARSQ’s interpretability validity [40]. Moreover, to examine the potential response bias between women with AAR experience during their past pregnancy within 1 year and those with a current pregnancy, DIF is a statistical approach used to identify unfair items for which different groups of respondents perform differently. The Chi-square parameter equality test has been calculated and if the *p* value is < 0.05, the presence of DIF has been verified [41].

### Concurrent and discriminate validity

Pearson correlation coefficients (*r*) between participant scores on the PARSQ and PSS were computed to provide evidence for the PARSQ’s concurrent validity. An *r* value of 0 indicates no association between two scores, a value greater than 0 implies a positive relation, and a value smaller than 0 indicates a negative association. The value of *r* between 0.1–0.3 represents a low-level relation between two scores; values between 0.3–0.5 indicate a medium-level relation, and values greater than 0.5 indicate a high relation [42]. Evidence for the PARSQ’s discrimination was supported by comparing the significant differences in the scores between the women with AAR and the healthy pregnant women. Independent t-test and Mann-Whitney U-test for small samples of a subgroup were adopted, and when the *p*-value is < 0.05, a significant difference exists between the two groups.

All statistical analyses were conducted with IBM SPSS 17.0 and ConQuest 3.0.1 [41]. Since the Rasch model does not require complete data to make estimates, and estimates still can be computed from the set of non-missing responses on the scale without imputing estimates or deleting any portion of the data [43], the responses of the filter items of the PARSQ were treated as missing-response data and left as a period (.) when conducting analysis using ConQuest.

## Results

### Characteristics of participants

Two hundred women with AAR experience (135 women with AAR experience during their past pregnancy within 1 year and 65 women with AAR experience during their current pregnancy) and 96 healthy women were included in this study. Since no unfair items of the PARSQ were indicated by the result of DIF (*p* = 0.267), we treated these two kinds of women with AAR experience as a whole group to test the psychometric properties of PARSQ.

As shown in Table 1, the majority of the women with AAR experience were younger than age 35 and had earned a bachelor’s degree. Most had full-time jobs, and approximately 80% had a family income of at least 40,000 Taiwan dollars per month (Table 1). Nearly 50% of the women’s AAR experience occurred at their first pregnancy, and 37% had hospitalization experience. Most of these women had the experience of taking tocolytic drugs for alleviating their TPTL symptoms, and Ritodrine (Yutopar) was the drug most commonly reported. Women’s AAR experience has been reported to occur at an average of 27.2 weeks of gestation. Aside from lacking activity restriction experience and treatments for pregnancy complications, there was no significant difference in the characteristics of the healthy pregnant women and the AAR women (Table 1).

**Table 1:** Participant characteristics

Characteristics	AAR women (*N* = 200) n (%)	Women with current AAR (*n* = 65) n (%)	Women with AAR within a year (*n* = 135) n (%)	Healthy women (*N* = 96) n (%)	*p* ^a^
Age (years)					.556
≤ 35	150 (75.0)	44 (67.7)	106 (78.5)	75 (78.1)	
36–40	47 (23.5)	20 (30.8)	27 (20.0)	19 (19.8)	
>40	3 (1.5)	1 (1.5)	2 (1.5)	2 (2.1)	
Educational status					.774
High school	21 (10.5)	2 (3.1)	19 (14.1)	2 (2.0)	
University/College	130 (65.0)	47 (72.3)	83 (61.5)	71 (74.0)	
Graduate School	49 (24.5)	16 (24.6)	33 (24.4)	23 (24.0)	
Employment					.175
Full time	145 (72.5)	48 (73.8)	97 (71.9)	63 (65.6)	
Part time	17 (8.5)	4 (6.2)	13 (9.6)	15 (15.6)	
Unemployed	38 (19.0)	13 (20.0)	25 (18.5)	18 (18.8)	
Household monthly income (Taiwan dollars)					.229
≤40,000	43 (21.5)	12 (18.5)	31 (23.0)	25 (26.0)	
40,001 ~ 80,000	75 (37.5)	26 (40.0)	49 (36.3)	41 (42.7)	
> 80,000	82 (41.0)	27 (41.5)	55 (40.7)	30 (31.3)	
Nulliparous	94 (47.0)	41 (63.1)	53 (39.3)	51 (53.1)	.190
Hospitalized for preterm labor: yes	74 (37.0)	19 (29.2)	55 (40.7)	–	
Tocolytics usage: yes	162 (81.0)	53 (81.5)	109 (80.7)	–	

^a^ The result of differences between women with AAR experience and healthy pregnant women was compared by Chi-square test

### Item analysis

The mean score range for all items was between 1.64 and 3.93, with the standard deviation (SD) ranging between 0.85 and 1.61. The mean scores of all items were within the mean of the whole scale ±1 SD (mean score of total scale = 3.04, SD = 1.41) (Table 2). In the extreme groups’ comparison, the differences between the top and bottom 27% of each item was between − 15.39 and − 3.91, and all reached significance (*p* < 0.001), indicating the PARSQ items were with good discrimination. Moreover, the Infit and Outfit of individual items ranged from 0.77–1.63 and 0.77–1.60, respectively, with most showing reasonable fit parameters except for items 6 and 23 (Table 2). After items 6 and 23 were deleted due to undesirable fit parameters, the remaining 23 items underwent further analysis.

**Table 2:** Item analysis for Prenatal Activity Restriction Stress Questionnaire (PARSQ) (*N* = 200)

Item of PARSQ	Mean (SD)	Extreme group comparison		RSM		Result
		High score/Low score group (n)	*t*-test ^d^	Infit	Outfit	
1. Feel troubled by not being able to go out to run errands	3.84 (1.20)	54/54	−9.15**	0.87	0.86	Keep
2. Feel troubled by not being able to prepare meals and do household chores	3.81 (1.22)	54/54	−10.07**	0.89	0.87	Keep
3. Feel troubled by not being able to take care of my other children (*n* = 106) ^a^	3.55 (1.34)	21/31	−4.87**	1.28	1.33	Keep
4. Feel distressed by having to rely on others to take care of myself	3.47 (1.23)	54/54	−10.10**	0.80	0.83	Keep
5. Feel distressed by having to rely on others to take care of my other children (*n* = 106) ^a^	3.66(1.18)	21/31	−3.91**	1.05	1.02	Keep
6. Worry about losing baby	2.66(1.61)	54/54	−7.55**	1.63	1.59	Delete
7. Worry about possible preterm birth	3.66(1.23)	54/54	−9.91**	0.86	0.83	Keep
8. Worry about baby’s development and health	3.93(1.08)	54/54	−9.45**	0.83	0.79	Keep
9. Worry about reduction in fetal movements	2.57(1.25)	54/54	−7.43**	1.05	1.04	Keep
10. Worry that the labor process may harm the baby	3.01(1.17)	54/54	−9.04**	0.85	0.84	Keep
11. Worry about baby care issues	3.17(1.15)	54/54	−6.97**	0.93	0.93	Keep
12. Feel impatient about physical discomfort, such as fatigue and difficulty falling asleep	3.72(1.18)	54/54	−6.32**	1.06	1.12	Keep
13. Feel impatient about my depressed mood	3.27(1.18)	54/54	−10.72**	0.81	0.82	Keep
14: Worry about the preterm labor signs continually appearing	3.56(1.18)	54/54	−.8.84**	0.86	0.93	Keep
15. Feel irritated with taking tocolytics (*n* = 162) ^b^	3.75(1.22)	41/52	−7.95**	1.02	1.07	Keep
16. Feel troubled by necessary physical activity; e.g. up and down stairs, taking bath and so on	3.28(1.22)	54/54	−9.62**	0.86	0.87	Keep
17. Worry about the strained spousal relationship	1.97(1.08)	54/54	−7.86**	1.02	1.03	Keep
18. Worry about relationships with other children becoming alienated (*n* = 106) ^a^	1.95(1.14)	21/32	−5.75**	1.03	0.93	Keep
19. Worry about relationships with other family members deteriorating	1.64(0.85)	54/54	−5.99**	0.91	0.97	Keep
20. Feel irritated with the unclear way of obtaining pertinent information on management of physical symptoms and coping with AAR	1.96(1.07)	54/54	−5.74**	0.98	0.98	Keep
21. Feel troubled with medical staff interactions	2.23(1.09)	54/54	−5.14**	1.08	1.25	Keep
22. Feel irritated with the frequent clinic visits	2.93(1.32)	54/54	−15.39**	0.77	0.77	Keep
23. Worry about losing my job (*n* = 162) ^c^	2.19(1.47)	39/39	−5.28**	1.57	1.60	Delete
24. Feel distressed with having to ask for leave from work for bed rest (*n* = 162) ^c^	3.76(1.36)	39/39	−4.38**	1.49	1.53	Keep
25. Feel distressed with the family’s financial strain	2.50(1.41)	54/54	−7.95**	1.31	1.27	Keep
Total scores	3.04 (1.41)					

*SD* standard deviation, *RSM* Rasch rating scale model, *AAR* Antepartum activity restriction

^a^ the responses of 106 parous women

^b^ the responses of 162 women with taking tocolytic-drug experiences

^c^ the responses of 162 women with full-or part- time job

^d^independent *t* test

**p < 0.001

### Dimensionality, reliability evidence of the PARSQ

To confirm the PARSQ’s structure, the two hypothesized models, namely the unidimensional and 4-dimensional models, were compared. The results showed that the 4-dimensional model was the better model, since its deviance was 113.73 less than that of the unidimensional model, and this value reached statistical significance in the Chi-square distribution with nine degrees of freedom (Table 3).

**Table 3:** Model comparison for Prenatal Activity Restriction Stress Questionnaire

Hypothesised models	Deviance	Parameters	Difference		*p* value
			Deviance	Estimated parameters	
Unidimensional	11,488.19	27	113.73	9	<0.001
Four-dimensional	11,374.46	36			

*p*-value was approximated using a Chi-square distribution table

The correlations among these 4 dimensions ranged from 0.638–0.845; meanwhile, the person-separation reliability of these dimensions ranged from 0.744–0.865, and the item-separation reliability was 0.993. These values indicate that the items in the 4 dimensions have adequate internal consistency and can separate respondents with different levels of stress of activity restriction. The Infit and Outfit MnSq of the remaining 23 items also demonstrated acceptable fit parameters under the 4-dimensional model (Table 4). The item difficulties are shown in Table 4. In dimension 1, the easiest item was item 1 (Feel troubled by not being able to go out to run errands), and the hardest was item 19 (Worry about relationships with other family members deteriorating). In dimension 2, the easiest item was item 8 (Worry about baby’s development and health), and the hardest was item 10 (Worry that the labor process may harm the baby). In dimension 3, the easiest item was item 12 (Feel annoyed by physical discomfort), and the hardest was item 16 (Feel troubled by necessary physical activity). And in dimension 4, the easiest item was item 24 (Feel distressed with having to ask for leave from work for bed rest), and the hardest was item 20 (Feel annoyed with the unclear way of obtaining pertinent information on management of physical symptoms and coping with AAR).

**Table 4:** Fit parameters and estimates of item difficulty for the PARSQ by dimensions (*N* = 200)

Dimension	Item number	Difficulty (logit)	Error	Infit MnSq	Outfit MnSq
1. Role function changes	1	−0.979	.060	0.92	0.88
	2	−0.931	.060	0.91	0.85
	3	−0.543	.068	1.18	1.20
	4	−0.563	.058	0.95	0.99
	5	−0.654	.068	1.13	1.20
	17	1.030	.062	1.15	1.14
	18	1.099	.072	1.08	0.98
	19	1.539^a^	.017	1.00	1.03
2. Fetal safety and health	7	−0.369	.050	0.94	0.88
	8	−0.675	.051	0.85	0.81
	9	0.674	.049	1.03	1.01
	10	0.261	.049	0.77	0.77
	11	0.109^a^	.099	0.96	0.98
3. Self-physical and -psychological care issues	12	−0.211	.050	1.08	1.07
	13	0.240	.049	0.82	0.81
	14	−0.049	.050	0.93	0.95
	15	−0.204	.053	1.14	1.12
	16	0.224^a^	.101	0.83	0.81
4. Socioeconomic and medical issues	20	0.771	.054	0.98	0.95
	21	0.440	.052	1.07	1.18
	22	−0.264	.050	0.93	0.94
	24	−1.105	.056	1.49	1.51
	25	0.158^a^	.106	1.34	1.30

^a^ indicates that the parameter of the last item on the dimension is constrained

### Interpretability evidence of the PARSQ

**Fig. 1:**
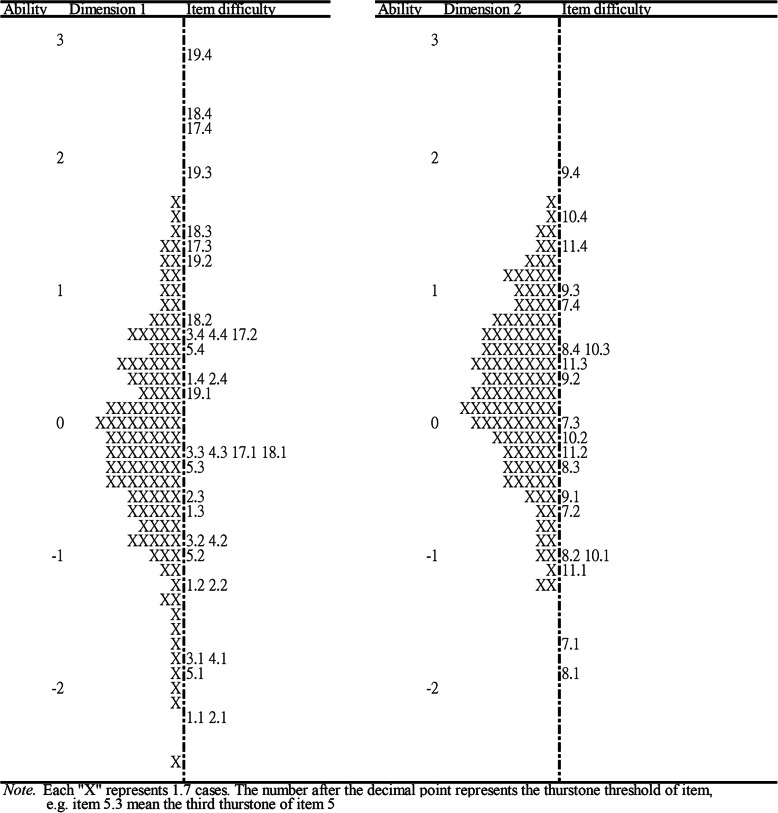
Item-person maps of dimensions 1 and 2 on the PARSQ

**Fig. 2:**
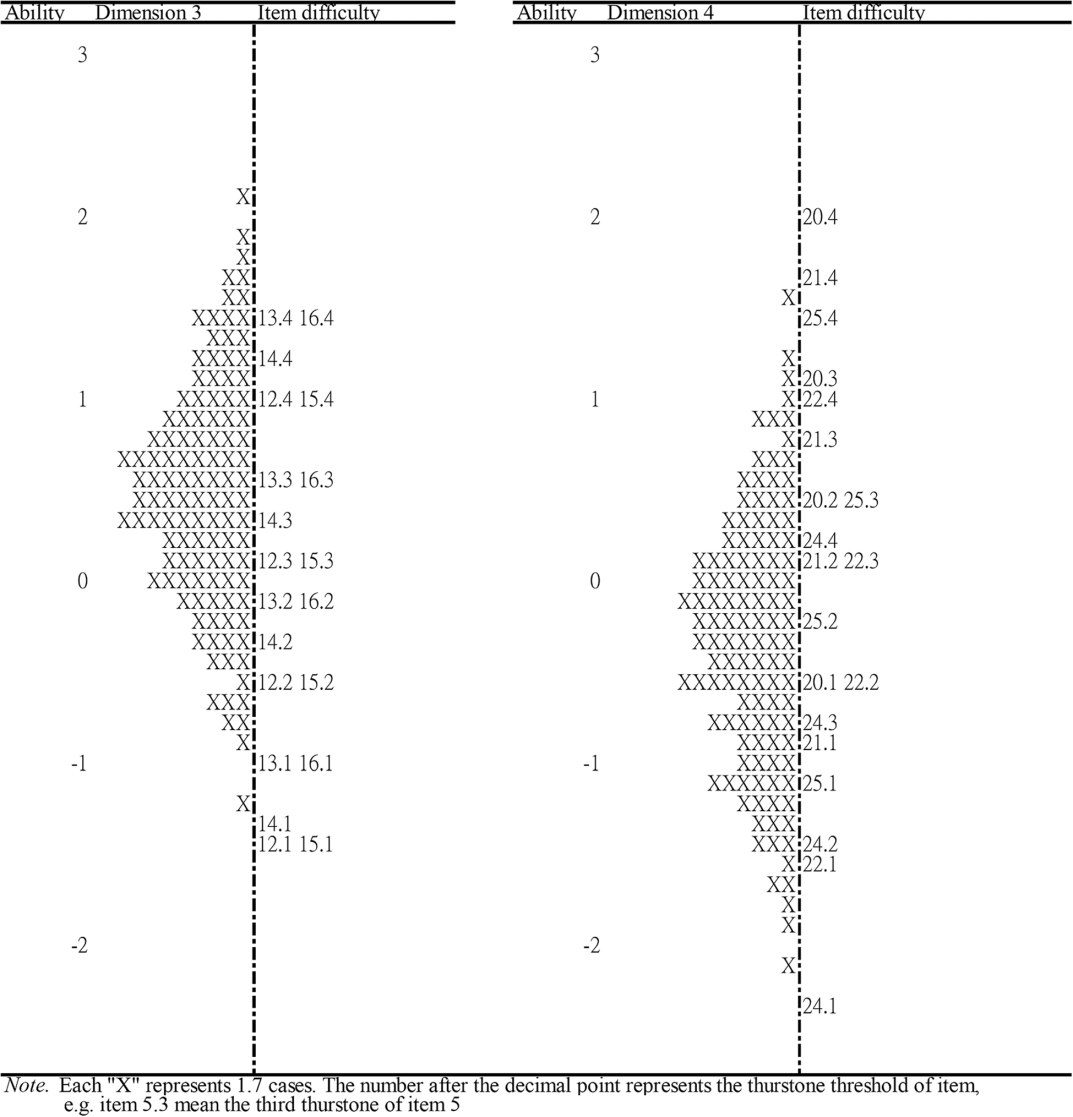
Item-person map of dimensions 3 and 4 on the PARSQ

The item-person maps by dimension were illustrated, and the item difficulties and person ability were estimated in the dimensions of PARSQ to assess whether the PARSQ is appropriate for the respondents (Figs. 1 and 2). For the item-person maps, the right side of the map showed the locations of item difficulty, while the left side demonstrated the distribution of respondent ability, which is marked with “X.” Items at the top displayed a higher difficulty than those at the bottom of the map, while higher locations for respondents indicated they had higher latent ability. Ideally, the difficulties of the items on the scale were expected to be spread across the ability range of all respondents; thus respondents with different abilities had items with corresponding difficulties that could accurately reflect their abilities.

**Fig. 3:**
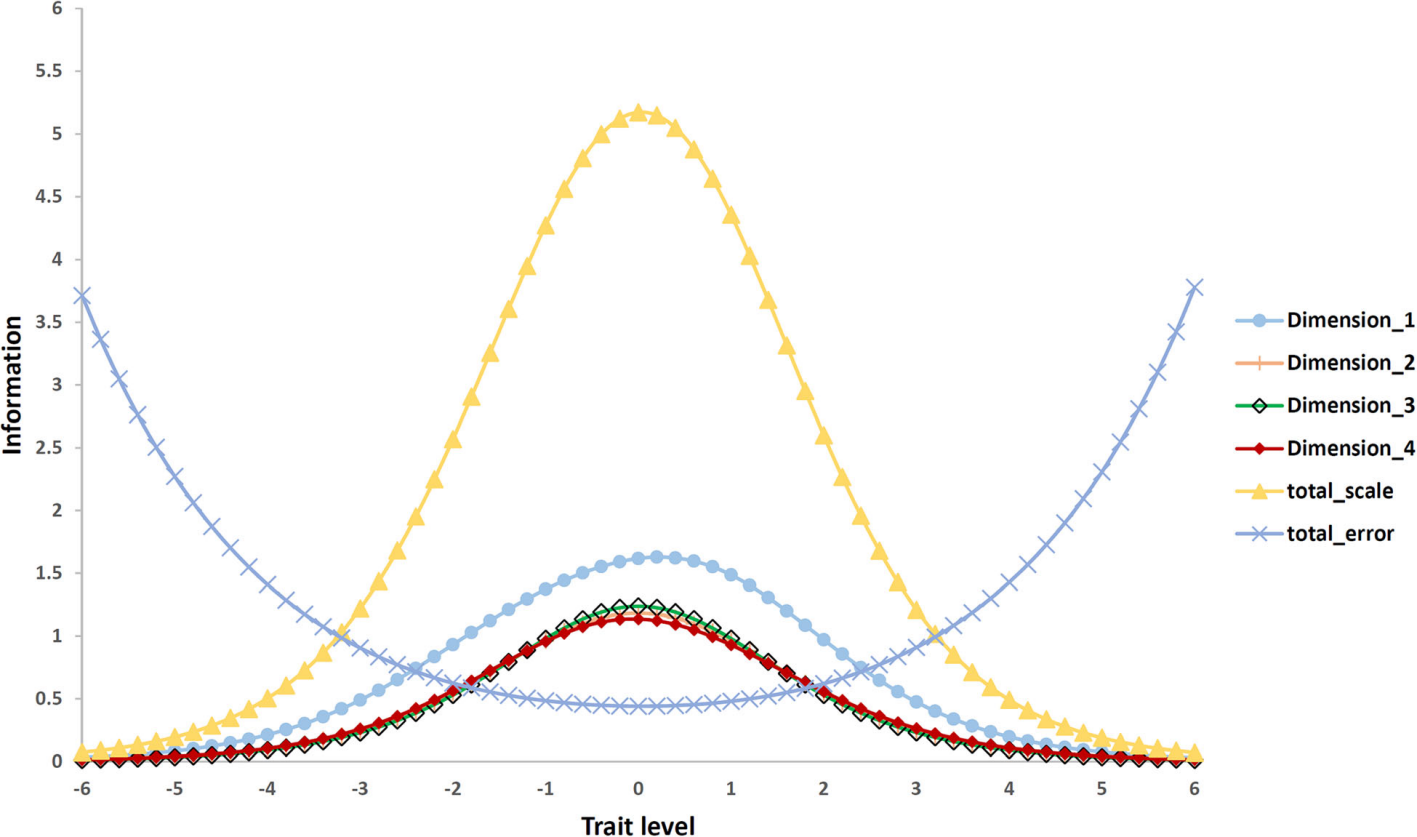
Test information curve for the full-scale and subscales of the PARSQ

As illustrated in Figs. 1 and 2, item difficulties were generally distributed among ±3 logits and the ability range of the respondents ranged from − 2.26–2.24 logits, 10 which indicated that the item difficulty was very close to the respondent ability and the items of the PARSQ can cover the distribution of most respondents’ ability. However, it should be noted that there was a lack of some equivalent-level items in dimension 3 to reflect the status of respondents with higher latent abilities. In addition, some items with similar difficulty also existed, such as the difficulty of item 3 being close to that of item 4 in dimension 1, and item 13 being close to that of item 16 in dimension 3. Moreover, the test information curves (Fig. 3) for the total and subscale of the PARSQ also demonstrated that the PARSQ provided the greatest amount of information for respondents who had abilities between ±3 logits. Above findings indicated the PARSQ had satisfied interpretive validity for women with AAR stress levels of ±3 logits.

### Concurrent and discriminant validity of the PARSQ

For verifying the concurrent validity of the PARSQ, the scores for the PARSQ and PSS of 65 pregnant women with activity restriction experience were analyzed. Results showed that women’s PARSQ scores positively correlated with their PSS scores (*r* = 0.348, *p* = 0.005), especially for dimensions 3 (*r* = 0.461, *p* < 0.001) and 4 (*r* = 0.382, *p* = 0.002). For examining the PARSQ’s discrimination, the total scale and subscale scores of the women with AAR experience and healthy pregnant women were compared. Since the women’s total scores on the PARSQ may be different due to their parity and employment status, subgroup analysis was conducted according to parity to confirm whether any difference in the PARSQ scores existed between the AAR women and the healthy pregnant women. The results indicated that the AAR women’s scores on the PARSQ total scale and subscales were all significantly higher than those of the healthy women regardless of parity and employment status (Table 5).

**Table 5:** Comparison of the difference in the PARSQ scores between the women with antepartum activity restriction experience and healthy pregnancy

Group / Dimensions		Fetal safety and health	Physical and psychological care issues ^c^	Role function changes	Socioeconomic and medical issues
Total	AAR (*N* = 200)	16.34 ± 4.14	13.82 ± 3.47	–	–
	Healthy (*N* = 96)	10.99 ± 3.65	8.45 ± 2.62		
	*p* ^a^	<.001	<.001		
Nulliparous	AAR (*n* = 94)	–	–	14.09 ± 4.34	–
	Healthy (*n* = 51)			8.41 ± 2.94	
	*p* ^a^			<.001	
Parous	AAR (*n* = 106)	–	–	24.44 ± 5.53	–
	Healthy (*n* = 45)			14.39 ± 4.05	
	*p* ^a^			<.001	
Employed	AAR (*n* = 160)	–	–	–	13.03 ± 3.9
	Healthy (*n* = 78)				7.64 ± 2.61
	*p* ^a^				<.001
Unemployed	AAR (*n* = 40)	–	–	–	9.14 ± 3.81
	Healthy (*n* = 18)				6.50 ± 3.05
	*p* ^b^				<.001

AAR = women with antepartum activity restriction experience. Dimension 1 as role function changes with item 1–5 and 17–19, Dimension 2 as fetal safety and health with item 7–11, Dimension 3 as physical and psychological care issues with item 12–16 and Dimension 4 as socioeconomic and medical issues with item 20, 21, 22, 24, 25

Data shown as Mean ± SD

^a^ independent *t*-tests

^b^ Mann-Whitney U-test

^c^ item 15 was excluded due to that no women with healthy pregnancy had this experience

## Discussion

Pregnancy stress has been proven to affect the health of the offspring [8]; it has therefore garnered much attention in recent years. As such, the stress of women undergoing AAR to prevent preterm birth should not be ignored since they have to face many challenges and must learn to live with the threat of preterm birth. To fill this need, this study developed and evaluated the psychometric properties of the PARSQ to assess the distress of women undergoing activity restriction during pregnancy. Finally, the PARSQ with 23 items measuring the 4 dimensions of distress (Role function changes, Fetal safety and health, Physical and psychological care issues, and Socioeconomic and medical issues) using 5-point Likert-type response scale was developed and confirmed to have appropriate validity and reliability.

Unlike previous scales, which were mostly developed based on the life situation of healthy pregnant women [16], the PARSQ was developed based on the life experience of medically high-risk women with AAR due to their TPTL symptoms. Thus, although there are other scales developed based on the context of medically high-risk women, like the High-Risk Pregnancy Stress Scale (HRPSS) [19], PARSQ has the preterm labor context-special items, such as issues of tocolytics treatment, relationship with important others, and employment. Furthermore, different from the previous 15 pregnancy-special stress instruments, all of whose psychometric properties were tested based on classical measurement theory [16], the item quality and item difficulty of the PARSQ were confirmed through the RSM. Therefore, each item of the PARSQ was fashioned with particular function and difficulty to detect the specific distresses of women with AAR, the features of which increase the convenience of its use in a time-limited clinical setting. For example, the simpler questions on each PARSQ dimension (like item 1 on dimension 1 and item 8 on dimension 2) can be performed first with the women, and then the more difficult questions can be given as needed. Moreover, since the equal interval measurement properties of the PARSQ were also verified by RSM, the total scores of the PARSQ are reasonable to compare differences in the degree of distress between groups. However, since the parity, treatment and employment status would affect women’s responses on the filter items and total scores on dimensions 1, 3, and 4, we suggest that subgroup analysis should be done according to the women’s parity, treatment, and employment status when conducting difference comparison.

Although advantages of the PARSQ had been demonstrated, some points and improvements should be of concern. First, although the PARSQ was developed based on comprehensive literature review and interviews with AAR women, apart from 4 items with unclear content that were deleted in content validity analysis, another 2 items (item 6: “losing baby” and item 23: “losing my job”), which unexpectedly showed misfit in the RSM, were also deleted. One possible reason for the misfit of item 6 may be related to the good accessibility of maternal healthcare under National Health Insurance in Taiwan [44], which could reduce a woman’s fear of losing her baby. Another reason could be the death taboo in Taiwanese culture, in which people are discouraged from talking about death as it may bring bad luck [45]. The content of item 6 could make the women associate it with stillbirth or fetus death; therefore, they would tend to conceal their true feelings and answer the question positively. The reason for the misfit of item 23 may be related to Taiwan’s Act of Gender Equality in Employment and Labor Standards Act, which gives women legal protection on their right to work during pregnancy [46]. Therefore, women who undergo activity restriction might not perceive much distress on item 23: “losing my job.” Second, to complete the PARSQ’s effectiveness, generating items with higher levels of stress difficulty in dimension 3 and modifying items with similar difficulties should be made in a subsequent study. Third, the PARSQ’s validity and reliability were primarily tested on a population of women with activity restriction due to TPTL. To expand the PARSQ’s usefulness, its validity and reliability could be further evaluated among women who are advised to restrict their activity to alleviate their pregnancy complications or improve the success of implantation [47]. Fourth, to understand the effect of stress of activity restriction on women’s pregnancy outcomes, the PARSQ’s predictive validity should be tested in the future. Finally, associations between the PARSQ and other biological measures could also be tested in future work to explore the influence of stress of activity restriction on pregnant women’s physiological health.

A main limitation of this study should be noted: non-pregnant women who had experienced AAR within the past year were included in this study. Even though no study revealed the different experiences with AAR between women who are pregnant and those who have already given birth, we still cannot exclude the impact of retrospective recall on the psychometric properties of the PARSQ. Retrospective recall can be influenced by a woman’s pregnancy outcome and may conflict with accurate recall [48]. Therefore, even though no DIF existed among items of the PARSQ between pregnant and non-pregnant AAR women, showing no difference in responses of these two groups on the PARSQ’s items, we cannot completely exclude the impact of retrospective recall on the psychometric properties of the PARSQ.

## Conclusion

Understanding the distress of women with should be a key component for developing appropriate prenatal care to *assist women* in coping with their situation to avoid the adverse consequences of the emotional burden on both maternal and offspring health. The developed PARSQ with 23 items and 4 dimensions was confirmed to have satisfied validity and reliability. Since the PARSQ is a content-based scale, it allows clinicians or researchers not only to characterize the stress levels experienced by women undergoing activity restriction, but it also offers a more tailored understanding of their distress during this specific period. Therefore, the PARSQ is a valuable measure that can be used as a foundation for healthcare providers to develop appropriate care for meeting women’s individual needs.

## Supplementary Information

**Additional file 1.** Prenatal Activity Restriction Stress Questionnaire (PARSQ) definitions and initial version for content validity review.

**Additional file 2.** Prenatal Activity Restriction Stress Questionnaire (PARSQ).

## Data Availability

The datasets are available from the corresponding author upon reasonable request.

## Abbreviations

AAR: Antepartum Activity Restriction
TPTL: Threatened preterm Labor
PARSQ: Prenatal Activity Restriction Stress Questionnaire
CTT: Classical Test Theory
I-CVI: Content validity index for each item
S-CVI: Content validity index for all scales
PSS: Perceived Stress Scale
RSM: Rasch rating scale model
Infit MnSq: Weighted mean-square fit statistics
Outfit MnSq: Unweighted mean-square fit statistics
DIF: Differential item functioning
