# Supplementary material for: Development and validation of the prenatal activity restriction stress questionnaire: a Rasch rating scale analysis
Source: BMC Pregnancy Childbirth. 2020 Oct 31;20:659. doi: 10.1186/s12884-020-03347-3 (PMC7603674; doi:10.1186/s12884-020-03347-3)
Supplement: Supplementary file 1 — Additional file 1. Prenatal Activity Restriction Stress Questionnaire (PARSQ) definitions and initial version for content validity review. [file 12884_2020_3347_MOESM1_ESM.docx]

Additional file 1. Prenatal Activity Restriction Stress Questionnaire (PARSQ) definitions and initial version for content validity review.

| **Construct** | **Dimensions** | **Definition** | **Initial item number and content** | | **Decision** | **Reordered item number** |
| --- | --- | --- | --- | --- | --- | --- |
| Prenatal Activity Restriction Stress | Role Function Changes | Distress or annoyance with changes in their family role and function stimulated by pregnancy complications and lifestyle changes | 16 | Feel troubled with not being able to go out and run some errands | Keep | 1 |
|  |  |  | 17 | Feel troubled with not being able to prepare meals and do household chores | Keep | 2 |
|  |  |  | 18 | Feel troubled with not being able to take care my other children | Keep | 3 |
|  |  |  | 19 | Feel distressed with having to rely on others to take care of myself | Keep | 4 |
|  |  |  | 20 | Feel distressed with having to rely on others to take care of my other children | Keep | 5 |
|  |  |  | 21 | Worry about the strained relationship with my husband | Keep | 17 |
|  |  |  | 22 | Worry about the alienated relationship with my other children | Keep | 18 |
|  |  |  | 23 | Worry about the deteriorated relationship with other family members | Keep | 19 |
|  | Fetal Safety and Health | Distress or worry about fetal safety and health derived by the pregnancy complications and lifestyle changes | 1 | Worry about losing baby | Keep | 6 |
|  |  |  | 2 | Worry about possible preterm birth | Keep | 7 |
|  |  |  | 3 | Worry about baby’s development and health | Keep | 8 |
|  |  |  | 10 | Worry about reduction in fetal movements | Keep | 9 |
|  |  |  | 4 | Worry about the labor process | Combine and modify | 10 |
|  |  |  | 5 | Worry about delivering an unhealthy baby |  |  |
|  |  |  | 6 | Worry about baby care issues | Keep | 11 |

Additional file 1. Prenatal Activity Restriction Stress Questionnaire (PARSQ) definitions and initial version for content validity review. (Continued)

| **Construct** | **Dimensions** | **Definition** | **Initial item number and content** | | **Decision** | **Reordered item number** |
| --- | --- | --- | --- | --- | --- | --- |
| Prenatal Activity Restriction Stress | Physical and Psycholo-gical Care Issues | Distress or annoyance with the issues of physical and psychological care stimulated by the pregnancy complications and lifestyles changes | 7 | Feel annoyed with physical discomfort, such as fatigue and difficulty falling asleep | Keep | 12 |
|  |  |  | 8 | Feel annoyed with my depressed mood | Keep | 13 |
|  |  |  | 9 | Worry about the preterm birth signs continually appearing | Keep | 14 |
|  |  |  | 10 | Feel annoyed with the tocolytics dosage | Combine and modify | 15 |
|  |  |  | 12 | Feel annoyed with the tocolytics frequency |  |  |
|  |  |  | 13 | Feel annoyed with taking different kinds of tocolytics |  |  |
|  |  |  | 14 | Feel annoyed with the side effects of tocolytics |  |  |
|  |  |  | 15 | Feel troubled about by necessary physical activities | Keep | 16 |
|  | Socio-  economic and Medical Issues | Distress or annoyance about employment, economics, and related medical activities stimulated by the pregnancy complications and lifestyle changes | 24 | Feel annoyed with the unclear way of obtaining pertinent information on management of physical symptoms and coping with activity restriction. | Keep | 20 |
|  |  |  | 25 | Feel troubled with medical staff interactions | Keep | 21 |
|  |  |  | 26 | Feel annoyed with frequent clinic visits | Keep | 22 |
|  |  |  | 27 | Worry about losing my job | Keep | 23 |
|  |  |  | 28 | Feel distressed with having to ask for leave from work for bed rest | Keep | 24 |
|  |  |  | 29 | Feel distressed with the family’s financial strain | Keep | 25 |
