# Supplementary material for: Development and validation of the prenatal activity restriction stress questionnaire: a Rasch rating scale analysis
Source: BMC Pregnancy Childbirth. 2020 Oct 31;20:659. doi: 10.1186/s12884-020-03347-3 (PMC7603674; doi:10.1186/s12884-020-03347-3)
Supplement: Supplementary file 2 — Additional file 2. Prenatal Activity Restriction Stress Questionnaire (PARSQ). [file 12884_2020_3347_MOESM2_ESM.docx]

Additional file

Prenatal Activity Restriction Stress Questionnaire (PARSQ)

Instructions:

**The following statements** are the distresses women encountered when they are confined at home or hospital for controlling their preterm birth symptoms during pregnancy. Please indicate how much you have experienced each of the following while you are confined at home or hospital for alleviating your preterm birth symptoms during pregnancy:

Please indicate your levels of distress by using the following scale. If any of the questions in items 3, 5, 15, 18, 23, and 24 are not applicable to your situation, please circle “not applicable (NA).”

Circle the “1”, if you **never** have such distress or concern

Circle the “2”, if you **rarely** have such distress or concern

Circle the “3”, if you **sometimes** have such distress or concern

Circle the “4”, if you **often** have such distress or concern

Circle the “5”, if you **always** have such distress or concern

| **Items** | | **Levels of distress or concern** | | | | | Not applicable  (NA) |
| --- | --- | --- | --- | --- | --- | --- | --- |
| 1 | Feel troubled by not being able to go out to run errands | 1 | 2 | 3 | 4 | 5 |  |
| 2 | Feel troubled about not being able to prepare meals and do household chores | 1 | 2 | 3 | 4 | 5 |  |
| 3 | Feel troubled about not being able to take care of my other children | 1 | 2 | 3 | 4 | 5 | NA |
| 4 | Feel distressed by having to rely on others to take care of myself | 1 | 2 | 3 | 4 | 5 |  |
| 5 | Feel distressed by having to rely on others to take care of my other children | 1 | 2 | 3 | 4 | 5 | NA |
| 6 | Worry about losing the baby | 1 | 2 | 3 | 4 | 5 |  |
| 7 | Worry about possible preterm birth | 1 | 2 | 3 | 4 | 5 |  |
| 8 | Worry about the baby’s development and health | 1 | 2 | 3 | 4 | 5 |  |
| 9 | Worry about reduction in fetal movements | 1 | 2 | 3 | 4 | 5 |  |
| 10 | Worry that the labor process may harm the baby | 1 | 2 | 3 | 4 | 5 |  |
| 11 | Worry about baby care issues | 1 | 2 | 3 | 4 | 5 |  |
| 12 | Feel impatient about my physical discomfort, such as fatigue and difficulty falling asleep | 1 | 2 | 3 | 4 | 5 |  |
| 13 | Feel impatient about my depressed mood | 1 | 2 | 3 | 4 | 5 |  |
| 14 | Worry about the preterm birth signs continually appearing | 1 | 2 | 3 | 4 | 5 |  |
| 15 | Feel irritated with taking tocolytics | 1 | 2 | 3 | 4 | 5 | NA |
| 16 | Feel troubled about necessary limitations on physical activities; e.g. going up and down stairs, taking bath, and so on | 1 | 2 | 3 | 4 | 5 |  |
| 17 | Worry about the strained relationship with my husband | 1 | 2 | 3 | 4 | 5 |  |
| 18 | Worry about the marginalized relationship with my other children | 1 | 2 | 3 | 4 | 5 | NA |
| 19 | Worry about the deteriorating relationship with other family members | 1 | 2 | 3 | 4 | 5 |  |
| 20 | Feel irritated with the unclear way of obtaining pertinent information on management of physical symptoms and coping with AAR | 1 | 2 | 3 | 4 | 5 |  |
| 21 | Feel troubled about medical staff interactions | 1 | 2 | 3 | 4 | 5 |  |
| 22 | Feel irritated with the frequent clinic visits | 1 | 2 | 3 | 4 | 5 |  |
| 23 | Worry about losing my job | 1 | 2 | 3 | 4 | 5 | NA |
| 24 | Feel distressed about having to ask for leave from work for bed rest | 1 | 2 | 3 | 4 | 5 | NA |
| 25 | Feel distressed with the family’s financial strain | 1 | 2 | 3 | 4 | 5 |  |
